# Interactive Media-Based Approach for an Exception From Informed Consent Trial Involving Patients With Trauma

**DOI:** 10.1001/jamasurg.2024.2147

**Published:** 2024-07-03

**Authors:** Shannon W. Stephens, Christy Carroll-Ledbetter, Sarah Duckert, Tanner Coffman, Margaret Nelson, Karen N. Brown, Joel Rodgers, Russell L. Griffin, Amy Suen, Jeremy Casey, Steven R. Sloan, Brahm Goldstein, Adam Joseph McClintock, Sara F. Goldkind, Luke Gelinas, Amanda E. Higley, Bellal A. Joseph, John B. Holcomb, Jan O. Jansen

**Affiliations:** 1 Center for Injury Science, University of Alabama at Birmingham, Birmingham; 2 Hematology Therapeutic Research Area, CSL Behring, King of Prussia, Pennsylvania; 3 Office of Institutional Review Board, University of Alabama at Birmingham, Birmingham; 4 Goldkind Consulting LLC, Potomac, Maryland; 5 Advarra, Columbia, Maryland; 6 Department of Surgery, University of Arizona, Tucson

## Abstract

**Importance:**

Exception From Informed Consent (EFIC) research requires community consultation (CC) and public disclosure (PD). Traditional methods of conducting CC and PD are slow, expensive, and labor intensive.

**Objective:**

To describe the feasibility and reach of a novel interactive, media-based approach to CC and PD and to identify the similarities and differences between trial sites in website views, survey responses, online community forum attendance, and opt-out requests.

**Design, Setting, and Participants:**

This survey study analyzed the CC and PD campaigns conducted for the TAP trial (Evaluation of BE1116 in Patients With Traumatic Injury and Acute Major Bleeding to Improve Survival), an EFIC trial of the early administration of prothrombin complex concentrate in patients with trauma. The CC and PD campaigns consisted of social media advertisements, linked websites, community surveys, and online community forums. These activities were coordinated from a central site and approved by a central institutional review board. This study focused on the first 52 of 91 TAP trial sites (level I trauma centers) in the US to have completed their CC and PD campaigns. Community members in the catchment areas of the participating trauma centers were targeted. Data analysis was conducted between October 2023 and February 2024.

**Exposure:**

Social media advertisements, surveys, and online community meetings conducted as part of the CC and PD campaign for the TAP trial.

**Main Outcomes and Measures:**

Social media campaign reach and engagement, web page views, survey results, online community forum attendance, and opt-out requests.

**Results:**

Fifty-two trial sites were approved for participant enrollment. Social media advertisements were displayed 92 million times, reaching 11.8 million individuals. The median (IQR) number of people reached in each location was 210 317 (172 068-276 968). Site-specific websites were viewed 144 197 times (median [IQR] viewings per site, 2984 [1267-4038]). A total of 17 206 fully or partly completed surveys were received, and survey respondents had a median (IQR) age of 40.1 (15-65) years and included 10 444 females (60.7%). Overall, 60.6% survey respondents said they would want to be entered into the trial even if they could not give consent, 87.7% agreed that emergency care research was necessary, and 88.0% agreed that the TAP trial should be conducted in their community. Online community forums were attended by a median (IQR) number of 38 (20-63) people. Four opt-out requests were received.

**Conclusions and Relevance:**

The interactive media-based approach to CC and PD for the ongoing TAP trial showed the feasibility and benefits of executing an efficient, coordinated, centrally run series of locally branded and geographically targeted CC and PD campaigns for a large EFIC study.

## Key Points

**Question:**

Is it possible for a single center to coordinate and execute the community consultation (CC) and public disclosure (PD) campaigns for a large Exception From Informed Consent (EFIC) clinical trial?

**Findings:**

In this survey study of an interactive media-based approach to access community members in the catchment areas of 52 trial sites, 11.8 million individuals were reached via social media advertisements, websites were viewed 144 197 times, and 17 206 individuals completed surveys.

**Meaning:**

Findings of this study suggest that conducting centralized CC and PD campaigns for EFIC trials is possible using an interactive, media-based approach to CC and PD can reach large numbers of individuals.

## Teaser

This survey study reports on the required community consultation and public disclosure activities for level I trauma centers participating in an Exception From Informed Consent (EFIC) trial in the US.

## Introduction

Clinical trials involving the acute care of patients with traumatic hemorrhagic shock, stroke, or cardiac arrest present numerous ethical challenges. Respecting participants and their autonomy, through the informed consent process and voluntary participation, is a cornerstone of ethical research. However, when patients have life-threatening conditions, they and their legally authorized representatives are usually unable to participate in the informed consent process without delaying potentially life-saving investigational treatment. This inability creates challenges to fulfilling the spirit of these ethical foundations.

In 1996, the US Department of Health and Human Services and the Food and Drug Administration issued guidelines for the execution of clinical studies in patients with emergent medical conditions. Federal regulations 21 CFR (Code of Federal Regulations) 50.24 and 45 CFR 46.101 outline the requirements for Exception From Informed Consent (EFIC) research.^1,2^ The key steps in these regulations are community consultation (CC) and public disclosure (PD).^3,4^

Traditional methods for CC include public meetings, community forums, newspaper and television advertisements, and telephone-based surveys. However, the ability of these processes to reach the target populations, effectively deliver information, and elicit useful feedback remains unclear.^5,6^ Additionally, these traditional methods for CC are time consuming, are expensive, and often yield little feedback, raising concerns that important populations were not reached.^6^

Over the past 2 decades, the Center for Injury Science at the University of Alabama at Birmingham has gained extensive experience in conducting social media–based CC and PD. However, recognizing the limitations of the social media–based approach, particularly with regard to eliciting meaningful feedback from communities, we have developed and refined a method that includes community surveys and online forums. This method, developed in consultation with an ethics consultant and the central Advarra Institutional Review Board (IRB), is the interactive, media-based approach.^7^ In this study, we applied this approach to the CC and PD campaigns for the TAP trial (Evaluation of BE1116 in Patients With Traumatic Injury and Acute Major Bleeding to Improve Survival),^8^ an 8000-patient, 120-center, international randomized clinical trial that evaluates the early use of 4-factor prothrombin complex concentrate in patients with trauma who are expected to require a large-volume blood transfusion.^9^ Approximately 90 of the TAP trial sites are located in the US, thus requiring CC and PD and making these CC and PD campaigns the largest to date.

The objective of this study was to describe the feasibility and reach of a novel interactive, media-based approach to CC and PD and to identify the similarities and differences between trial sites in terms of website views, survey responses, online community forum attendance, and opt-out requests. Findings of this study may facilitate further development of CC and PD methods for multicenter EFIC trials.

## Methods

All US-based CC and PD campaigns were designed and coordinated by the Center for Injury Science, in conjunction with the TAP trial sites, and were approved by the Advarra IRB. This analysis focused on the first 52 of 91 US sites to have completed their CC and PD campaigns. Forty-nine sites also required local IRB approval or acknowledgment of the CC and PD campaigns. At the conclusion of each campaign, the Center for Injury Science staff prepared a 30-page site-specific report, which was then submitted to the Advarra IRB to gain approval to start enrollment. Advarra IRB approved the present survey study; informed consent was not required because the work was an EFIC study.

### Social Media Advertisements

We created social media advertisements on Facebook and Instagram, 2 of the leading social media platforms that allow advertisements to be targeted to specific geographic areas and age groups. These advertisements were linked to site-specific websites providing detailed information about the TAP trial, EFIC research in general, and how to opt out.^10^ The advertisements and the wording on the websites were largely standardized and agreed to by the sites. Social media advertisements were targeted to individuals aged 15 years or older who lived either within a specified radius (typically 50 miles) of the participating trauma center (denoting that center’s catchment area for patients arriving directly from the scene of an incident) or in specific zip code areas known to have a high incidence of trauma.

### Institution-Specific Linked Websites

We created institution-specific websites to provide details about the TAP trial, Frequently Asked Questions (FAQs) and answers, EFIC research in general, how to opt out, and how to contact the local investigator. We also included a page describing the online community forums.

### Community Surveys

The community surveys were conducted using a cloud-based survey software platform (Qualtrics; Qualtrics). Designed by the Center for Injury Science, the surveys could be accessed on each site’s website or directly on the platform. Surveys could be accessed by anyone but were specifically targeted to individuals residing in counties within the catchment area of participating trauma centers. All free-text comments received as part of the community surveys were included in the reports submitted to the Advarra IRB.

### Online Community Forums

Each site conducted at least 4 online community meetings. Two of these meetings were general meetings; open to the public; and advertised using IRB-approved social media posts, local public service announcements, flyers placed at local emergency departments and clinics, and other processes (eg, departmental websites). The other 2 meetings were targeted to support groups, such as the Trauma Survivors Network or Mothers Against Drunk Driving. Support groups were selected by local research teams. Meetings were conducted using video conferencing technology (either Zoom [Zoom Video Communications Inc] or Microsoft Teams [Microsoft Corporation]), jointly arranged by site research staff and the Center for Injury Science, and chaired by the local site’s principal investigator. A trained mediator from the Center for Injury Science attended meetings and, using a template, recorded attendance, favorable and unfavorable opinions expressed, and questions asked and answered. During meetings, the polling feature of Microsoft Teams or Zoom was used to solicit feedback from attendees. Data from the meetings were included in the reports submitted to the Advarra IRB.

### Statistical Analysis

Data on social media reach were obtained from the 2 social media platforms used. We used a data analytics platform (Google Analytics; Google LLC) to quantify visitor information to each web page. Survey responses were downloaded from the Qualtrics platform. Data on community forum attendance and opt-out requests were extracted from prospectively maintained records. Statistical analysis was descriptive, with results presented as numbers and proportions as well as medians and interquartile ranges (IQRs) and was conducted between October 2023 and February 2024 using Microsoft Excel (Microsoft Corporation).

## Results

### Social Media Reach and Engagement

**Figure 1.**
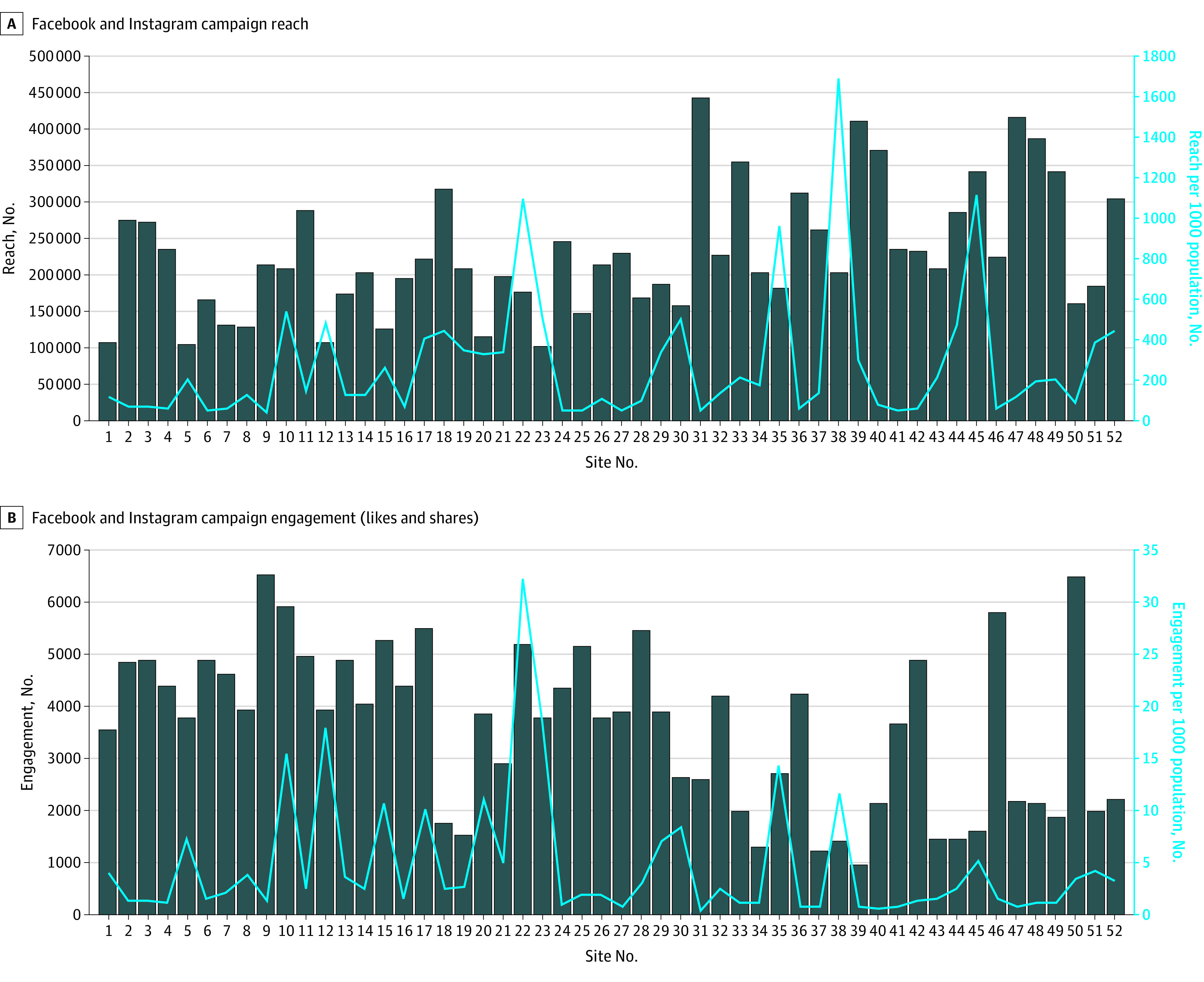
Social Media Reach and Engagement

The advertisements on social media were displayed a total of 92 million times across all catchment areas of the 52 sites and reached a total of 11.8 million unique individuals. All 52 sites were approved for enrollment by the Advarra IRB. Figure 1A shows the number of individuals reached by the advertisements and the number of individuals reached per 1000 population by site. The number of individuals reached in each location ranged from 101 190 to 440 327, with a median (IQR) of 210 317 (172 068-276 968). Catchment populations of the sites, as defined by their trauma centers, ranged from 119 800 to 10 520 655 (median [IQR], 1 711 169 [557 424-3 400 902]), and the number of individuals reached per 1000 population ranged from 38 to 1685 (median [IQR], 134 [67-355]).

Figure 1B shows the number of individuals by site who engaged with the social media advertisements by liking or sharing. The median (IQR) number of engagements was 3843 (2111-4860) individuals. Again, when indexed to catchment population size, the median (IQR) number of engagements was 2.3 (1.1-5.0) per 1000 population.

### Web Page Views

The institution-specific websites were viewed 144 197 times (median [IQR] viewings per site, 2984 [1267-4038]). The median (IQR) number of page viewings per 1000 population was 1.5 (0.7-3.5). The eFigure in Supplement 1 shows the number of views that the main landing pages, FAQ pages, EFIC description pages, and opt-out information pages received by site. The main landing pages were viewed most frequently (median [IQR] viewings, 2936 [1250-3924]), followed by the community meeting pages (median [IQR] viewings, 13 [5-25]), opt-out information pages (median [IQR] viewings, 12 [7-19]), FAQ pages (median [IQR] viewings, 10 [5-20]), and EFIC description pages (median [IQR] viewings, 9 [4-16]). There was a discernible temporal pattern, in that websites that have been active and able to be accessed for longer were viewed by more people.

### Survey Question Replies

**Figure 2.**
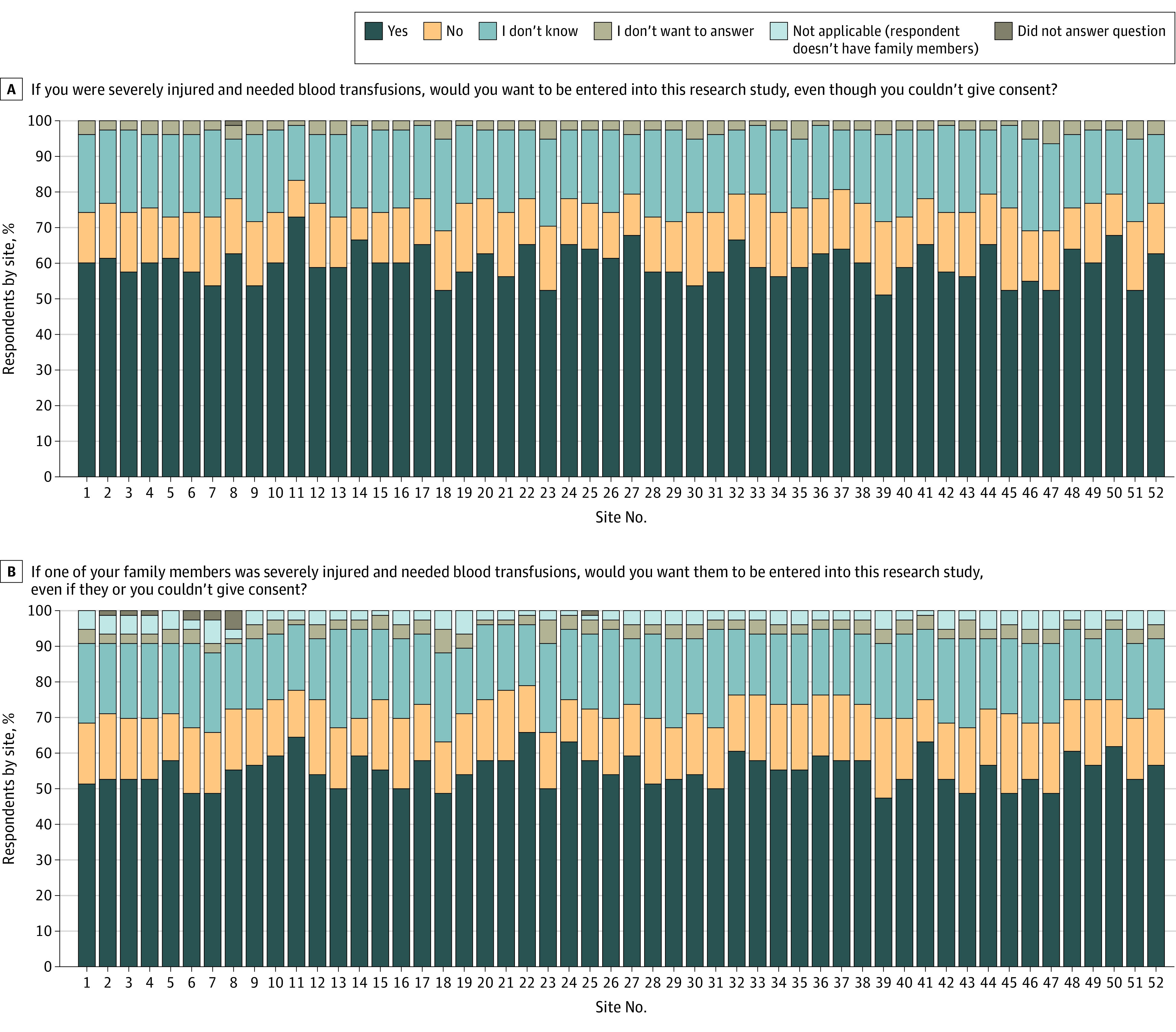
Replies to Survey Questions on the Desire to Enroll in the Study A. Respondents were also informed that “We would still try to get consent for continued participation after you are stable and your legally authorized representative can be identified.” B. Respondents were also informed that “We would still try to get consent for continued participation after their arrival to the hospital.”

In total, 17 206 fully or partly completed surveys were received. Survey respondents comprised 10 444 females (60.7%) and 6762 males (39.3%), with a median (IQR) age of 40.1 (15-65) years. For the question “If you were severely injured and needed blood transfusions, would you want to be entered into this research study, even though you couldn’t give consent?” 60.6% of participants responded with yes, 15.2% with no, 21.1% with “I do not know,” and 0.3% with “I do not want to answer”; 0.04% did not answer the question. The median (IQR) number of surveys completed by site was 314 (304-334), and the site-specific proportions are shown in Figure 2A. The median (IQR) proportion of yes responses was 60.1% (57.3%-63.7%), demonstrating a high degree of consistency across all sites.

In response to the question “What is the reason for your concern?” 30.9% of participants cited “fears about negative effects of the study,” 38.2% said “patients should not lose the right to provide consent for themselves,” 6.2% had other reasons, 19.5% did not know, and 5.2% did not want to answer.

Figure 2B shows the responses to the question “If one of your family members [rather than the respondent themselves] was severely injured and needed blood transfusions, would you want them to be entered into this research study, even if they or you couldn’t give consent?” Overall, 55.8% of participants responded with yes and 16.6% declined to answer. The median (IQR) proportion of yes responses across all 52 sites was 55.5% (52.2%-58.6%). The reasons for a no response differed, with 34.0% citing “fear about negative effects of the study” and 40.6% claiming “patients should not lose the right to provide consent for themselves.”

**Figure 3.**
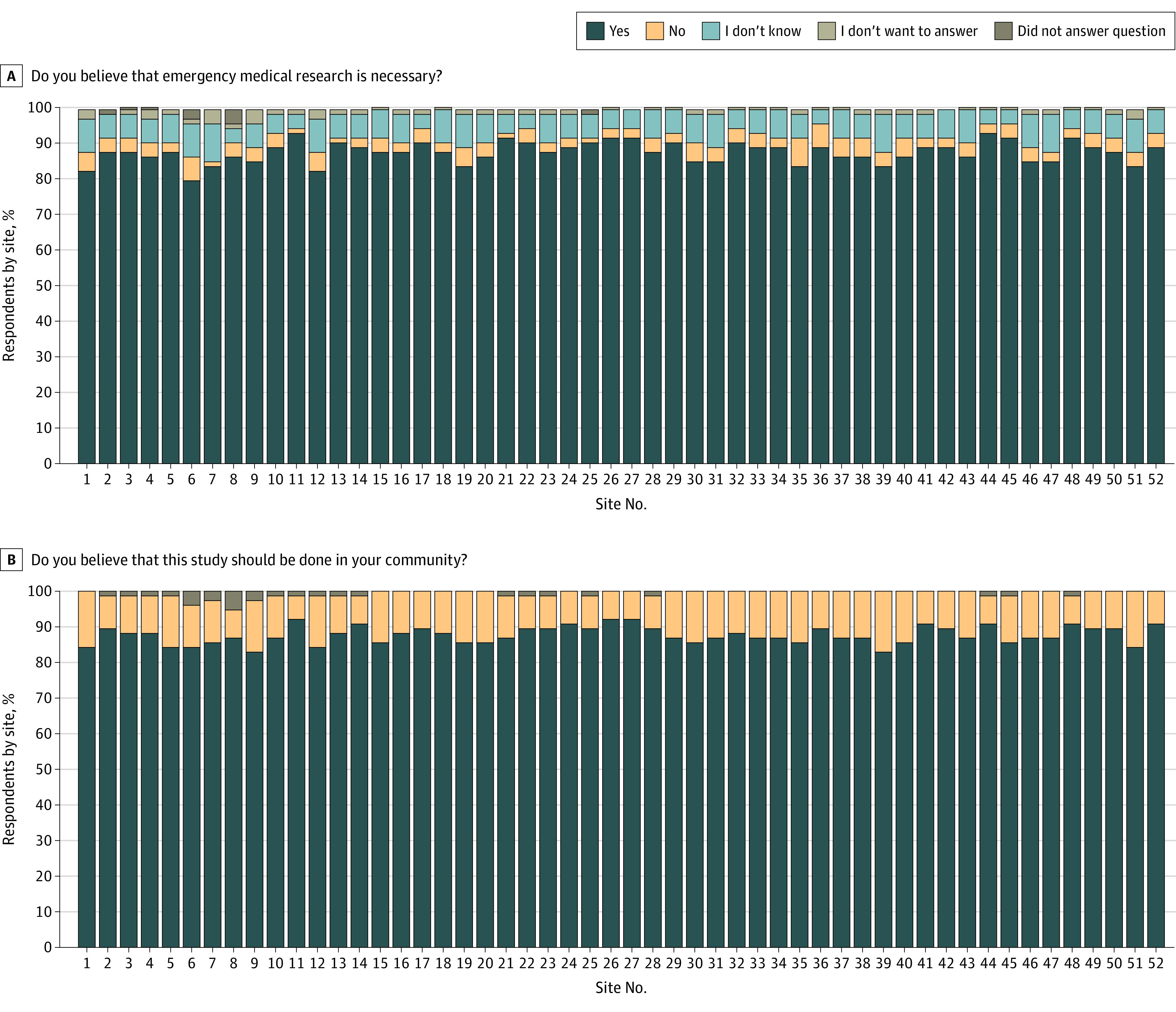
Replies to Survey Questions on the Necessity of Emergency Medical Research

Figure 3A shows the responses to the question “Do you believe that emergency medical research is necessary?” Overall, 87.7% of participants responded with yes, and the responses were consistent across sites (median [IQR], 87.6% [85.7%-89.4%]). Similarly, Figure 3B shows responses to the question “Do you believe that this study should be done in your community?” A total of 88.0% agreed. The responses were also consistent across all sites (median [IQR], 87.4% [86.3%-89.7%]) (Figure 3D).

### Online Community Forum Attendance and Opt-Out Requests

**Figure 4.**
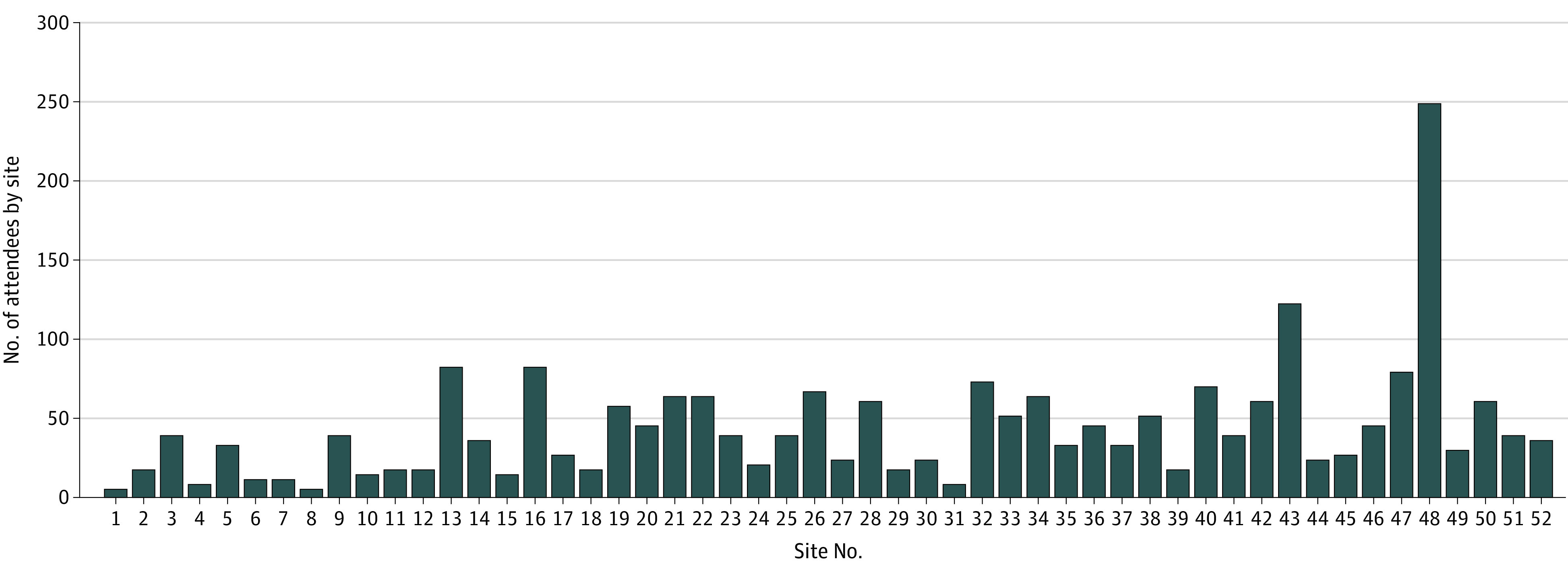
Online Community Forum Attendance

Figure 4 shows the total number of individuals who attended online community forums by site. The median (IQR) was 38 (20-63) cumulative attendees. We received a total of 4 opt-out requests from 4 different centers.

## Discussion

The CC and PD campaigns for the TAP trial were, to our knowledge, the largest such campaigns conducted to date. Previous large multicenter EFIC studies have typically relied on individual sites to design and conduct their own EFIC trials. The TAP trial differs in that, although each site’s CC and PD campaign was geographically targeted and locally branded, the template used for each campaign was the same, and the entire effort was designed and coordinated centrally by the Center for Injury Science.

Over half of US adults receive news and learn about current events from social media.^11^ In the US, 235.1 million people aged 18 years or older (88.6% of the population) spend an average of 145 minutes per day on social media. Using social media as the primary means of community outreach far surpasses more traditional methods.^11^ Our results are consistent with findings of studies that demonstrate the wide reach of social media and the ability to engage community members in defined geographic areas.^7,12,13,14^ The trial advertisements were displayed 92 million times; 11.8 million unique individuals were reached, and 17 206 people completed surveys about this trial. This type of broad PD is critical because it allows interested individuals to engage further. Conversely, individuals may be content with having been informed about the study and choose to not be involved with the consultation process or simply liking or sharing the social media post.

The results of the present study also revealed that the proportion of individuals reached varied when considered as a proportion of the catchment population. This variation could be due to inaccuracies in estimating an area’s catchment population (some inaccuracies are associated with commuters) and to the algorithms used by social media sites for the geographic targeting of advertisements. There was also a change in health-related advertising procedures on Facebook in April 2023, which may explain the lower engagement seen on the right of Figure 1B.

The interactive media-based approach we developed is more comprehensive than a previous method, which relied on social media alone.^7^ In particular, with the new approach, there is a greater opportunity for individuals to convey their views, which is one of the tenets of CC.

Approximately two-thirds of respondents said they would want to be enrolled, or would want a relative to be enrolled, in the TAP trial if they were brought to the trauma center injured and lacked the capacity to provide informed consent. The proportion was slightly smaller among respondents who wanted a relative to be enrolled in the trial if they lacked the capacity to consent. These results are consistent with the results of other published work.^15^ When asked for possible reasons that they would not want to be enrolled or would not want a relative to be enrolled, the most common responses were “fear about negative effects of the study” and “patients should not lose the right to provide consent for themselves.” The latter answer indicates either limited comprehension of the subject matter, as the questions specifically stated “even though you couldn’t give consent,” or poor wording of the question.

A considerably greater proportion of participants agreed that emergency care research was necessary and that the TAP trial should be run in their community compared with the proportion of participants who would want to be included in the study or would want their relatives to be included in the study. This response may reflect a person’s individualism, favoring freedom of action over what is deemed best for the community. Alternatively, it may be associated with the order in which the questions were asked, with individuals developing greater comfort with the subject matter as they progressed through the survey. This situation could be avoided by random ordering of questions in future surveys. Despite only two-thirds of people indicating that they would want to be enrolled or would want relatives to be enrolled, the actual number of opt-out requests received was small.

We found that, despite extensive advertising, participation in online forums was limited. However, limited participation was also the case when CC was primarily conducted in person. Harvin et al^6^ found that traditional community meetings, when conducted concurrent with an existing standing meeting, yielded fewer than 20 attendees. Face-to-face engagement may be even more problematic now, because of the large number of requests that individuals receive (whether by social media, email, or otherwise) and the resulting consultation fatigue. Completing surveys, although also subject to fatigue, is easier than attending community meetings for many people because doing so is more convenient and perhaps less daunting.

The interactive media-based approach is appealing to participating research sites, particularly those that have not previously conducted EFIC research. Setting up a CC and PD campaign, especially without prior experience, is difficult, time consuming, and expensive, with an attendant risk of site-selection bias and variability.^16^ To date, all TAP trial sites have received approval to start enrollment from the central IRB. Additionally, a centrally run CC and PD campaign ensures that, regardless of social, economic, demographic, or geographic inequalities, participating communities receive the same opportunities for consultation and engagement and that the results of these activities are comparable.

Executing CC and PD campaigns for many sites, compiling the results, and preparing reports are labor intensive. The Center for Injury Science employs 4 full-time staff to conduct this work, including a social media expert, a website creator, a data coordinator, and a moderator for the online forums. This expenditure needs to be incorporated into budgets. However, the overall cost of an interactive media-based approach to CC and PD is still less than that of traditional campaigns.^6^

### Limitations

There are limitations to this approach, including its reliance on social media, which disregards individuals without access to the internet. However, the number of such individuals is small (approximately 93% of the US population has internet access^17,18^), and the reach and efficiency of the approach outweigh this disadvantage, especially when considering the low penetration rate of traditional methods of CC and PD. Questions have been raised regarding who uses the 2 social media platforms used in this study and whether younger people would prefer, for example, other platforms (eg, TikTok, Snapchat, or X). A previous study found that Facebook-based campaigns reach a sample of the community broadly representative of the age and sex of the population as a whole.^12^ Data on race and ethnicity are no longer collected by social media companies because these data have been abused in the past. Lack of such information makes it difficult to characterize the sample that has been reached for the CC and PD campaign. Social media platforms are constantly evolving, as shown by the recent transformation of Twitter to X, and CC and PD approaches similarly need to adapt.

Another limitation is how community and representative should be defined, as explored in a previous work.^4,7^ As with traditional informed consent, it is difficult to evaluate the intended participant’s comprehension and understanding of the CC and PD materials. Some studies suggest that decreasing the length and complexity of consent materials is associated with improved overall comprehension of the study details, but this association remains unproven in EFIC research.^19^ Furthermore, federal regulations do not define the components of adequate CC and PD, such as the number of individuals reached or surveyed or the proportion of individuals who would agree to participate. Thus, regulators and investigators must work together.

## Conclusions

This survey study of an interactive media-based approach to CC and PD for the ongoing TAP trial revealed the feasibility and benefits of an efficient, coordinated, centrally run series of locally branded and geographically targeted CC and PD campaigns for a large EFIC study.

## Notes

Supplement 1. **eFigure.** Web Page Views

Supplement 2.
Nonauthor Collaborators

Supplement 3.
Data Sharing Statement
