# Supplementary material for: Interactive Media-Based Approach for an Exception From Informed Consent Trial Involving Patients With Trauma
Source: JAMA Surg. 2024 Jul 3;159(9):1051–8. doi: 10.1001/jamasurg.2024.2147 (PMC11223059; doi:10.1001/jamasurg.2024.2147)
Supplement: Supplement 1. — eFigure. Web Page Views [file jamasurg-e242147-s001.pdf]

Supplementary Online Content

Stephens SW, Carroll-Ledbetter C, Duckert S, et al; for the TAP Study Group. Interactive media-based approach for an Exception From Informed Consent trial involving patients with trauma. *JAMA Surg*. Published online July 3, 2024. doi:10.1001/jamasurg.2024.2147

eFigure. Web Page Views

This supplemental material has been provided by the authors to give readers additional information about their work.

© 2024 Stephens SW et al. *JAMA Surgery*.

eFigure 1. Web page views

A. Main Landing page

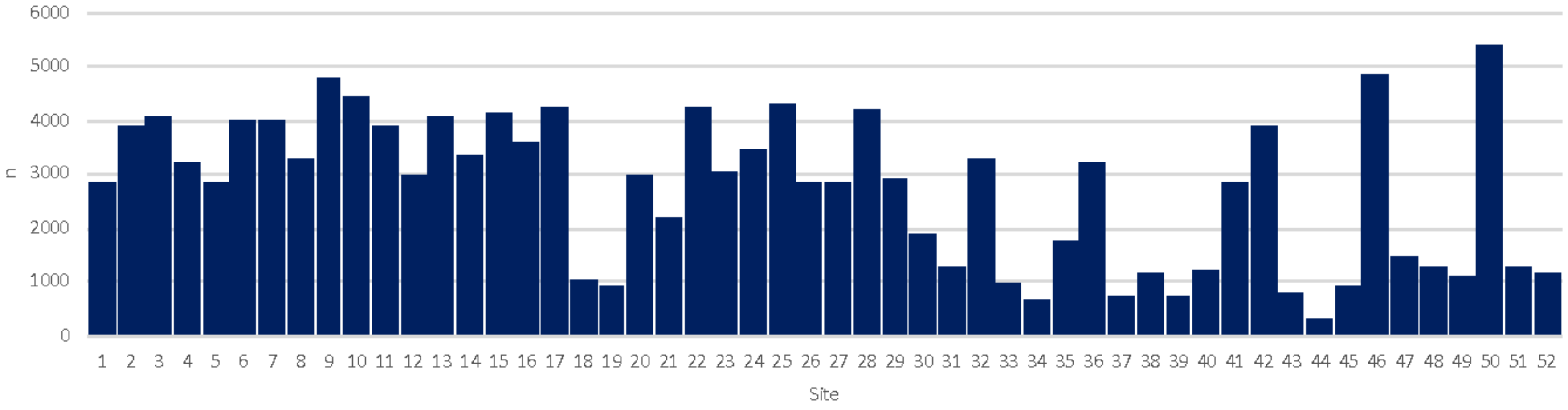

B. FAQ page

© 2024 Stephens SW et al. *JAMA Surgery*.

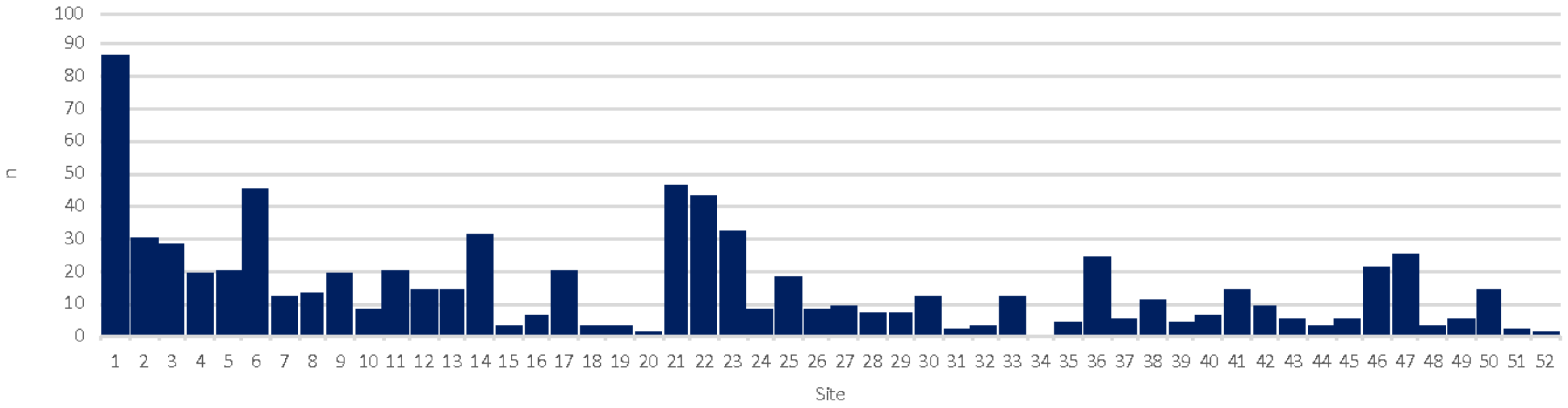

C. EFIC page

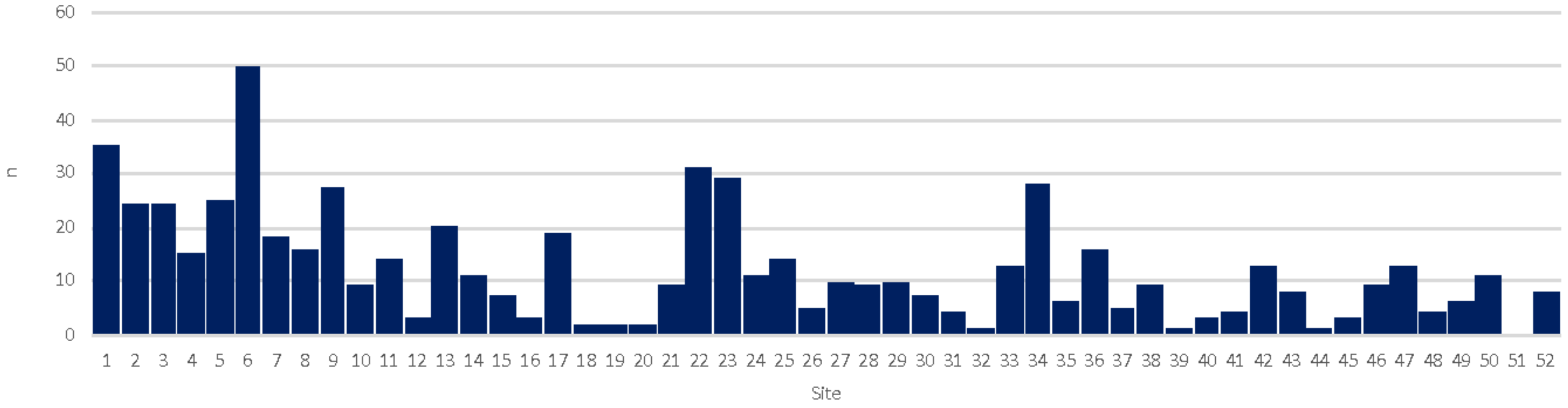

D. Opt out page

© 2024 Stephens SW et al. *JAMA Surgery*.

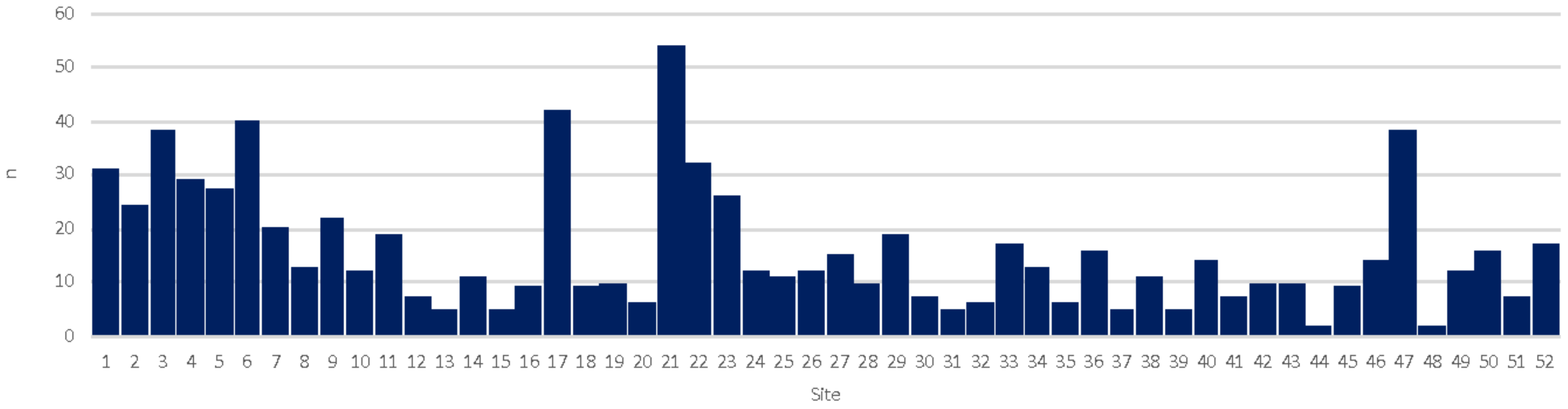

© 2024 Stephens SW et al. *JAMA Surgery*.
