# Supplementary material for: Interactive Media-Based Approach for an Exception From Informed Consent Trial Involving Patients With Trauma
Source: JAMA Surg. 2024 Jul 3;159(9):1051–8. doi: 10.1001/jamasurg.2024.2147 (PMC11223059; doi:10.1001/jamasurg.2024.2147)
Supplement: Supplement 2. — Nonauthor Collaborators [file jamasurg-e242147-s002.pdf]

Supplemental Online Content: Nonauthor Collaborators

*Indicates required information. Only first name, last name, and suffix will appear in PubMed.

*Group Name(s): TAP Investigators							
***First Name and Middle Initial(s)**	***Last Name**	***Suffix (eg, Jr, III)**	Academic Degrees	Institution	Location (city, state/province, country)	Role or Contribution, eg, chair, principal investigator	Group (if more than 1 Group listed in the byline) and/or Subgroup (eg, Steering Committee)
Amer A.	Afaneh		MD, FACS	Mercy St. Vincent Medical Center	Toledo, OH	Principal Investigator	N/A
Suresh	Agarwal		MD, FACS	Duke University	Durham, NC	Principal Investigator	N/A
Tanya	Anand		MD, MPH	University of Arizona	Tucson, AZ	Principal Investigator	N/A
Jeffrey H.	Anderson		MD	Temple University Hospital	Philadelphia, PA	Principal Investigator	N/A
Saman	Arbabi		MD	Harborview Medical Center	Seattle, WA	Principal Investigator	N/A
Dennis W.	Ashley		MD	Atrium Health Navicent-Mercer University School of Medicine	Macon, GA	Principal Investigator	N/A
James	Bardes		MD	West Virginia University	Morgantown, WV	Principal Investigator	N/A
Elizabeth R.	Benjamin		MD,PHD,FACS	Grady Memorial Hospital - Emory School of Medicine	Atlanta,GA	Principal Investigator	N/A
Paul M.	Bjordahl		MD FACS	Sanford USD Medical Center	Sioux Falls, SD	Principal Investigator	N/A
Grant V.	Bochicchio		MD,MPH, FACS	Washington University in St Louis School of Medicine	St. Louis, MO	Principal Investigator	N/A
Nikolay	Bugaev		MD	Tufts Medical Center	Boston, MA	Principal Investigator	N/A
Charles C	Butts		MD, FACS	University of South Alabama	Mobile, AL	Principal Investigator	N/A
Jeremy W.	Cannon		MD, SM	Perelman School of Medicine at the University of Pennsylvania	Philadelphia, PA	Principal Investigator	N/A
Margo	Carlin		DO	Texas Tech University Health Sciences Center	El Paso, TX	Principal Investigator	N/A

Supplemental Online Content: Nonauthor Collaborators

*Indicates required information. Only first name, last name, and suffix will appear in PubMed.

***First Name and Middle Initial(s)**	***Last Name**	***Suffix (eg, Jr, III)**	Academic Degrees	Institution	Location (city, state/province, country)	Role or Contribution, eg, chair, principal investigator	Group (if more than 1 Group listed in the byline) and/or Subgroup (eg, Steering Committee)
Jeffrey A	Claridge		MD	Metro Health Medical Center, Case Western Reserve University	Cleveland, OH	Principal Investigator	N/A
Raul	Coimbra		MD, PHD	Riverside University Health Sytsem	Moreno Valley, CA	Principal Investigator	N/A
Daniel B	Cox		MD	University of Alabama at Birmingham	Birmingham, AL	Principal Investigator	N/A
Alisa	Cross		MD	University of Oklahoma Health Science Center	Oklahoma City, OK	Principal Investigator	N/A
Warren C	Dorlac		MD	Medical Center of the Rockies	Loveland, CO	Principal Investigator	N/A
Brian E.	Driver		MD	Hennepin County Medical Center	Minneapolis, MN	Principal Investigator	N/A
Joseph	Dubose		MD	University of Texas at Austin Dell Medical School	Austin, TX	Principal Investigator	N/A
Raj R.	Gandhi		MD, PhD, FACS, FCCM	Burnett School of Medicine at TCU	Fort Worth, TX	Principal Investigator	N/A
Richard	George		MD	Summa Health	Akron, OH	Principal Investigator	N/A
Michael D.	Goodman		MD	University of Cincinnati	Cincinnati, OH	Principal Investigator	N/A
Jason	Hoth		MD	Wake Forest School of Medicine	Winston Salem, NC	Principal Investigator	N/A
Charles	Hu		MD	Chandler Regional Medical Center	Chandler, AZ	Principal Investigator	N/A
Uroghupatei P.	Iyegha		MD, FACS	Regions Hospital	St. Paul, MN	Principal Investigator	N/A
Lewis E.	Jacobson		MB, ChB	Ascension St. Vincent Indianapolis	Indianapolis, IN	Principal Investigator	N/A

*Indicates required information. Only first name, last name, and suffix will appear in PubMed.

***First Name and Middle Initial(s)**	***Last Name**	***Suffix (eg, Jr, III)**	Academic Degrees	Institution	Location (city, state/province, country)	Role or Contribution, eg, chair, principal investigator	Group (if more than 1 Group listed in the byline) and/or Subgroup (eg, Steering Committee)
Donald H.	Jenkins		MD	UT Health San Antonio	San Antonio, TX	Principal Investigator	N/A
Kyle J.	Kalkwarf		MD	University of Arkansas for Medical Sciences	Little Rock, AK	Principal Investigator	N/A
John P.	Kepros		MD	Honor Health	Scottsdale, AZ	Principal Investigator	N/A
Matthew E.	Kutcher		MD,MS	University of Mississippi Medical Center	Jackson, MS	Principal Investigator	N/A
Christine M.	Leeper		MD,MS	University of Pittsburgh	Pittsburgh, PA	Principal Investigator	N/A
Stephanie N.	Lueckel		MD,ScM	Rhode Island Hospital, Warren Alpert Medical School at Brown University	Providence, RI	Principal Investigator	N/A
David A.	Machado-Aranda		MD	Ronald Reagan – UCLA Medical Center (also University of California, Los Angeles can be suffice)	Los Angeles, California	Principal Investigator	N/A
Sarah D.	Majercik		MD, MBA	Intermountain Medical Center	Salt Lake City, UT	Principal Investigator	N/A
Adrian A.	Maung		MD	Yale School of Medicine	New Haven, CT	Principal Investigator	N/A
Katherine E.	McKenzie		DO	Jamaica Hospital Medical Center	Queens, NY	Principal Investigator	N/A
Jonathan P.	Meizoso		MD, MSPH, FACS	University of Miami Miller School of Medicine, Ryder Trauma Center, Jackson Memorial Hospital	Miami, FL	Principal Investigator	N/A
Jeffry	Nahmias		MD, MHPE	University of California	Orange, CA	Principal Investigator	N/A

Supplemental Online Content: Nonauthor Collaborators

*Indicates required information. Only first name, last name, and suffix will appear in PubMed.

***First Name and Middle Initial(s)**	***Last Name**	***Suffix (eg, Jr, III)**	Academic Degrees	Institution	Location (city, state/province, country)	Role or Contribution, eg, chair, principal investigator	Group (if more than 1 Group listed in the byline) and/or Subgroup (eg, Steering Committee)
Terence	O'Keeffe		MB,ChB,BSc, MsPH	Augusta University	Augusta, GA	Principal Investigator	N/A
Yuri	Rojavin		MD	Capital Health	Trenton, NJ	Principal Investigator	N/A
Samuel W	Ross		MD	Atrium Health - Carolinas Medical Center	Charlotte, NC	Principal Investigator	N/A
Navdeep S.	Samra		MD	Louisiana State University Health Shreveport	Shreveport, LA	Principal Investigator	N/A
Martin	Schreiber		MD	Oregon Health & Science University	Portland, OR	Principal Investigator	N/A
Thomas J	Schroeppel		MD	Univeristy of Colorado Health	Colorado Springs, CO	Principal Investigator	N/A
Timothy J.	Stevens		MD	Northeast Georgia Medical Center	Gainesville, GA	Principal Investigator	N/A
Jordan A.	Weinberg		MD	St. Joseph's Hospital and Medical Center	Phoenix, AZ	Principal Investigator	N/A
Thomas M	Wertin		MD	Valleywise Health Medical Center	Phoenix, AZ	Principal Investigator	N/A
Chad T.	Wilson		MD, MPH	Baylor College of Medicine	Houston, TX	Principal Investigator	N/A
Jonathan	Wisler		MD	The Ohio State University Wexner Medical Center	Columbus, OH	Principal Investigator	N/A
Khaled	Zreik		MD	Sanford Health	Sioux Falls, SD	Principal Investigator	N/A
