# Supplementary material for: Interactive Media-Based Approach for an Exception From Informed Consent Trial Involving Patients With Trauma
Source: JAMA Surg. 2024 Jul 3;159(9):1051–8. doi: 10.1001/jamasurg.2024.2147 (PMC11223059; doi:10.1001/jamasurg.2024.2147)
Supplement: Supplement 3. — Data Sharing Statement [file jamasurg-e242147-s003.pdf]

Data Sharing Statement

Stephens. Interactive Media-Based Approach for an Exception From Informed Consent Trial Involving Patients With Trauma. *JAMA Surg*. Published July 03, 2024.
doi:10.1001/jamasurg.2024.2147

Data
Data available: No

Additional Information
Explanation for why data not available: The CC/PD campaigns are ongoing and data continue to be collected from other sites.
